# Sleep patterns, sluggish cognitive tempo, and daytime sleepiness – a commentary on Fredrick et al. (2022)

**DOI:** 10.1111/jcpp.13693

**Published:** 2022-09-06

**Authors:** Dena Sadeghi‐Bahmani, Serge Brand

**Affiliations:** ^1^ Department of Psychology Stanford University Stanford CA USA; ^2^ Center for Affective, Stress and Sleep Disorders (ZASS), Psychiatric Clinics (UPK) University of Basel Basel Switzerland; ^3^ Division of Sport Science and Psychosocial Health, Department of Sport, Exercise, and Health University of Basel Basel Switzerland; ^4^ Sleep Disorders Research Center Kermanshah University of Medical Sciences Kermanshah Iran; ^5^ Substance Abuse Prevention Research Center Kermanshah University of Medical Sciences Kermanshah Iran; ^6^ Department of Psychiatry, School of Medicine Tehran University of Medical Sciences Tehran Iran

## Abstract

Fredrick et al. (*Journal of Child Psychology and Psychiatry*, 2022) showed in their cross‐sectional and observational study that higher Sluggish Cognitive Tempo (SCT) traits were associated with more impaired subjective and objective sleep parameters. Importantly, data were gathered from adolescents and their parents, thus, enhancing the validity of the findings. In addition, the observed pattern of associations was unrelated to ADHD traits, age, sex, medication, or pubertal development. In the present commentary, we acknowledge the scientific value and practical and clinical implications of these findings. For future studies, we propose seven research avenues, which might help to further clarify the neurophysiological, psychological, and behavioral associations between SCT traits and sleep patterns.

This issue of the *Journal of Child Psychology and Psychiatry* carries the report by Fredrick, Yeaman, Yu, Langberg, and Becker (2022) into the associations between Sluggish Cognitive Tempo (SCT) traits and dimensions of quantitative and qualitative sleep patterns among adolescents. Frederick and colleagues recruited 302 adolescents (mean age: 13.17 years; 44.7% females) with (*n* = 162) and without ADHD (*n* = 140) and their parents to take part in this multi‐method and multi‐informant study. Multi‐method refers to the fact that both objective (actigraphs) and subjective (questionnaires) were collected; multi‐informant refers to the fact that both adolescents and their parents reported SCT traits and sleep data on adolescent children's sleep and daytime sleepiness. Results showed that adolescents' higher self‐reported SCT trait scores were associated with shorter sleep duration and later sleep onset, where these were assessed via both self‐ratings and actigraphy. Higher scores for SCT traits were also associated with more difficulties initiating and maintaining sleep, as rated by parents, along with more sleep–wake problems and daytime sleepiness (adolescent ratings). Overall, the beauty of the study is that Fredrick et al. (2022) provide sufficient and convincing evidence that irrespective of the diagnosis of ADHD and irrespective of source of information (questionnaire; actigraphs self‐report; parents), stronger SCT traits are associated with impaired sleep patterns assessed both quantitatively and qualitatively.

In the present commentary, we would like to acknowledge and emphasize the extent to which the work of Fredrick et al. (2022) expands upon the current literature on the associations between SCT traits and adolescents' sleep patterns. We also propose some avenues for future investigations into this issue. Further initiatives are justified, because it appears that the concept of SCT remains under‐recognized as a health issue among children and adolescents; the domain of sleep in individuals with SCT has received modest research attention while individuals with SCT and ADHD need different treatment options (Becker et al., 2022). When untreated or inadequately treated, children and adolescents with ADHD are at increased risk of mental and behavioral issues in adulthood (Millenet et al., 2018), including higher odds of substance use (Molina et al., 2018), and difficulties in emotion regulation (Stern et al., 2020).

The strengths of the study of Fredrick et al. (2022) are as follows: (a) The authors provide strong evidence that among adolescents, poor sleep is associated with elevated SCT traits; (b) This association persists even after controlling for sex, age, ADHD status, medication use, and pubertal development; (c) The pattern of results is particularly robust and reliable, as both objective and subjective data and from child and parent reports were gathered. (d) In parallel to impaired sleep, daytime sleepiness increased.

For future studies, we suggest the research agenda may be enriched in the following seven ways:

First, by nature, the cross‐sectional study design precludes understanding the direction of causal association. While it is conceivable that higher SCT traits may lead to poor sleep and higher daytime sleepiness, the opposite direction of influence is also possible. Among about 10‐year‐old children with diagnosed ADHD, a 12‐week sleep‐training program has been shown to improve subjective sleep patterns and scores on dimensions of emotion regulation, ADHD‐related behavior and social interaction (Keshavarzi et al., 2014). Given this, we propose that future studies on the associations between SCT and sleep should be at least longitudinal observational studies, and preferably intervention studies specifically intended to improve sleep.

Second, while Fredrick et al.'s (2022) study nicely confirms *that* there is an association between SCT traits and poor sleep, it remains unclear, *why*. As noted elsewhere (Sadeghi‐Bahmani & Brand, 2022), insomnia as a proxy for poor sleep has been found to be associated with an overall higher default state of cortical hyperarousal as early as adolescence. Compared to adults with no insomnia, adults with insomnia display an increased structural covariance in cortical thickness between sensory and motor regions and an increase in default‐mode network activity. Results using functional magnetic resonance image (fMRI) have indicated that lack of sleep negatively modulates the emotional brain response, and suggested an impaired connection between the amygdala and prefrontal regions (Yoo, Gujar, Hu, Jolesz, & Walker, 2007). Future studies on the associations between SCT traits and sleep might introduce sleep‐EEG‐measurements or even imaging to investigate the neuronal processes underlying such associations.

Third, there is good evidence that dimensions of dysfunctional emotion regulation and poor sleep are associated among children, adolescents, and adults (Palmer & Alfano, 2017). Consequently, future studies might investigate whether and if so to what extent improving dimensions of emotion regulation and SCT traits also improve sleep.

Fourth and relatedly, children and adolescents with ADHD display high comorbidity rates for symptoms of anxiety and depression (Mayer et al., 2022); similar patterns have also been observed for individuals with SCT (Smith, Zald, & Lahey, 2020). Given this, future studies should assess symptoms of depression and anxiety as possible confounders blurring or even accentuating the associations between SCT traits and sleep.

Fifth, from the perspective of positive psychology, higher mental toughness traits in adolescents and adults have been shown to be associated with higher subjective and objective sleep patterns (Sadeghi‐Bahmani & Brand, 2022). Given this, future studies on SCT might investigate to what extent dimensions of mental toughness positively counteract SCT traits.

Sixth, some adolescents report a circadian shift toward a relative preference to go to bed later, when compared to their childhood, and a relative preference to wake‐up later in the morning, when compared to their childhood. Also, Fredrick et al. (2022) observed that poor sleep was also associated with a relative preference for eveningness. Future studies in the field of SCT traits should introduce dimensions of bed‐time‐related procrastination (Herzog‐Krzywoszanska & Krzywoszanski, 2019) to identify whether dysfunctional pre‐sleep cognitions and sleep‐delaying activities such as excessive use of media and social network sites (SNS) mediate the relation between SCT traits and poor sleep.

Seventh, cross‐sectional, longitudinal, and interventional studies have shown that regular physical activity and exercising are protective factors for mental health and sleep quality (Kalak et al., 2012). With this in mind, future studies on SCT might assess patterns of physical activity and exercising as buffers to counterbalance symptoms of depression, anxiety, and poor sleep among children and adolescents with SCT traits.

Overall, Fredrick et al. (2022) showed that SCT traits and poor sleep are associated among adolescents, and as such, the authors provided striking evidence but above all an excellent starting point for further investigation via longitudinal and interventional studies of the extent to which manipulating both sleep and physical activity could have a positive influence on sleep patterns, including daytime sleepiness, and daytime behavior among individuals with elevated SCT. The results reported by Fredrick et al. (2022) are also of clinical and practical importance, as they not only help clinicians, practitioners but also adolescents and their parents, teachers and policymakers to draw more attention to SCT traits, as SCT is neither listed in the DSM‐5 (American Psychiatric Association, 2013) nor introduced in the ICD‐11 (World Health Organization, 2019).
